# ATP-Binding Cassette (ABC) Transporter Genes in Plant-Parasitic Nematodes: An Opinion for Development of Novel Control Strategy

**DOI:** 10.3389/fpls.2020.582424

**Published:** 2020-11-20

**Authors:** Rinu Kooliyottil, Koushik Rao Gadhachanda, Nejra Solo, Louise-Marie Dandurand

**Affiliations:** ^1^Citrus Budwood Registration Program, Division of Plant Industry, Florida Department of Agriculture and Consumer Services, La Crosse, FL, United States; ^2^Department of Entomology, Plant Pathology and Nematology, University of Idaho, Moscow, ID, United States; ^3^Independent Researcher, Cheshire, CT, United States

**Keywords:** plant parasitic nematodes, resistance, xenobiotic metabolism, ABC transporters, gene silencing

## Introduction

Nematodes and plants have interacted for millions of years. Over the years, plant-parasitic nematodes (PPNs) have developed sophisticated mechanisms to overcome the immune response from plants, being able to establish successful parasitism in susceptible host plants. Today, nematodes have complex feeding structures along with other highly adaptive features, which suit their environment (Ali et al., 2017). Along with nematode evolution, plants have also adapted to recognize changes in pathogens for continued effective defense response. Initial contact with the PPNs triggers immune response in the host plant system which includes the release of toxic molecules. To put a bridle on this immune response, PPNs trigger pivotal cytoprotective mechanisms, such as antioxidant and detoxification pathways (Gillet et al., 2017). Mechanisms of these pathways have been studied in PPNs, and the specific genes involved have been targeted for gene silencing research in view of developing novel control measures (Gillet et al., 2017; Qiu et al., 2019). However, one of the important group of proteins involved in detoxification pathways known as ATP-binding cassette (ABC) transporters have not been studied until recently in PPNs. This opinion article focuses on the current knowledge and prospects of ABC transporters in PPNs.

## Plant–Nematode Interactions

Plants use a set of induced and constitutive strategies to protect themselves against pathogens. The protective measures are activated when pathogen-derived compounds called pathogen-associated molecular pattern (PAMPs) are recognized. In resistant plants, PAMP perception activates pattern-triggered immunity (PTI), which initiates signals that facilitate resistance to the growth of pathogens (Jones and Dangl, 2006). In plant-parasitic nematodes, small molecules called ascarosides, an evolutionarily conserved family of nematode pheromones, are known to induce microbe-associated molecular pattern (MAMP)-triggered immunity (Manosalva et al., 2015). An important ascaroside in plant-parasitic nematodes, ascr#18, was reported earlier to induce defense signaling pathways in *Arabidopsis*, tomato, potato, and barley to viral, bacterial, oomycete, fungal, and nematode infections (Manosalva et al., 2015). In resistant plants, the nucleotide-binding site–leucine-rich repeat (NB-LRR) proteins recognize the pathogen effectors which leads to effector-triggered immunity (ETI). Effector-triggered immunity directs one of the most effective plant defense mechanisms called the hypersensitive response (HR) (Bigeard et al., 2015), whereby a few cells surrounding the ingressing pathogen or pest die to ward off the pathogen. Potato cyst nematode *Globodera pallida* secretes protein RBP-1, which is known to induce defense responses, including cell death typical of HR through the NB-LRR protein Gpa2 (Sacco et al., 2009). Some early signs of HR are rapid influxes of free calcium (Ca^2+^), production of the reactive oxygen species (ROS), nitric oxide, and changes in the phytohormone production (Garcia-Brugger et al., 2006; Lozano and Smant, 2011). Among several important roles during the plant defense response, rapid influxes of Ca^2+^ are considered to be crucial for the activation of the NADPH oxidase found on the membrane of the plant cell (Kadota et al., 2015). Oxidases produce extracellular ROS, which initiate a cascade of events leading to an oxidative burst (Lozano and Smant, 2011). An oxidative burst along with the production of ROS is also an important part of the plant defense, since ROS create a cytotoxic environment for the pathogen or pest and also act as signaling molecules for local and systemic defense responses (Rosso, 2009; Gillet et al., 2017).

## Xenobiotic Metabolism to Counteract Plant Resistance

Pathogens and pests, including plant-parasitic nematodes, use antioxidant, and detoxification pathways in order to protect themselves and establish successful infections. Pathogens with endoparasitic lifestyles, which are exposed to the plant defense response for a considerable amount of time during their life cycle, use this mechanism very efficiently to overcome the host resistance (Robertson et al., 2000). The response of nematodes to the oxidative stress is predominantly regulated by transcription factors, *daf-16* and *skn-1*, which are also important for the survival of nematodes in their pre-parasitic stage (Gillet et al., 2017). These transcription factors are responsible to activate various antioxidant and detoxifying pathways, which function to avoid formation of the highly toxic ROS, to control and neutralize levels of ROS, as well as to prevent cellular damage due to oxidative stress (Callahan et al., 1988; Rosso, 2009; Gillet et al., 2017). In the nematode antioxidant pathway, *daf-16* and *skn-1* act together to regulate the expression of genes such as superoxide dismutase (SOD) and catalase (CTL), genes that encode glutathione peroxidases (GPX) and peroxiredoxin (PRDX) (Gillet et al., 2017). Xenobiotic/endobiotic detoxification pathway, also mediated by *daf-16* and/or *skn-1*, is activated against internal or external toxic compounds (Gillet et al., 2017) and has a role in the detoxification and excretion of these compounds (Lindblom and Dodd, 2009). Basso et al., 2020 used *in planta* RNAi technology to silence the *daf-16* and *skn-1* transcription factors and achieved significant resistance to root knot nematode *Meloidogyne incognita*. Silencing of *daf-16* and *skn-1* also resulted in the downregulation of important genes involved in detoxification pathway of the nematode.

There are three phases of xenobiotic metabolism. Phase I, which mainly involves cytochrome P450s, makes xenobiotics and endobiotics more soluble, while phase II is a detoxification step. In this phase, enzymes such as uridine dinucleotide phosphate glucuronosyltransferases (UGT) and glutathione S-transferases (GST) catalyze conjugate formation of xenobiotics and endobiotics with glutathione, amino acids, acetate, sulfate, propionate, or phosphate marking them for excretion (Kurutas, 2015; Laing et al., 2015). Most commonly this involves conjugation to glutathione (GSH), which is a tripeptide (γ-Glu-Cys-Gly) that has a major role in the processes of detoxification and redox buffering. In its reduced form, it acts as a nucleophile that attacks electrophilic carbon, nitrogen, or sulfur atom on the toxic non-polar compound (Edwards et al., 2000; Islam et al., 2017). Together with other antioxidants, such as ascorbate, α-tocopherol, and cysteine, it is an important aspect of non-enzymatic protection against oxidative stress (Kurutas, 2015). In animals, including nematodes, phase III involves excretion of these conjugates by ABC transporters, which do not belong to the family of detoxifying enzymes (Lindblom and Dodd, 2009). ABC transporters play a major role in the pumping of xenobiotic and endogenous metabolites through extra- and intracellular membranes, which helps to reduce the cellular concentrations of toxic compounds. Recent developments in xenobiotic metabolism in plant-parasitic nematodes give evidence of a multi-layered strategy using various effectors in a systematic way to protect from host-derived xenobiotic compounds (Espada et al., 2016; Lilley et al., 2018).

## ABC Transporters in Plant-Parasitic Nematodes

**Figure 1:**
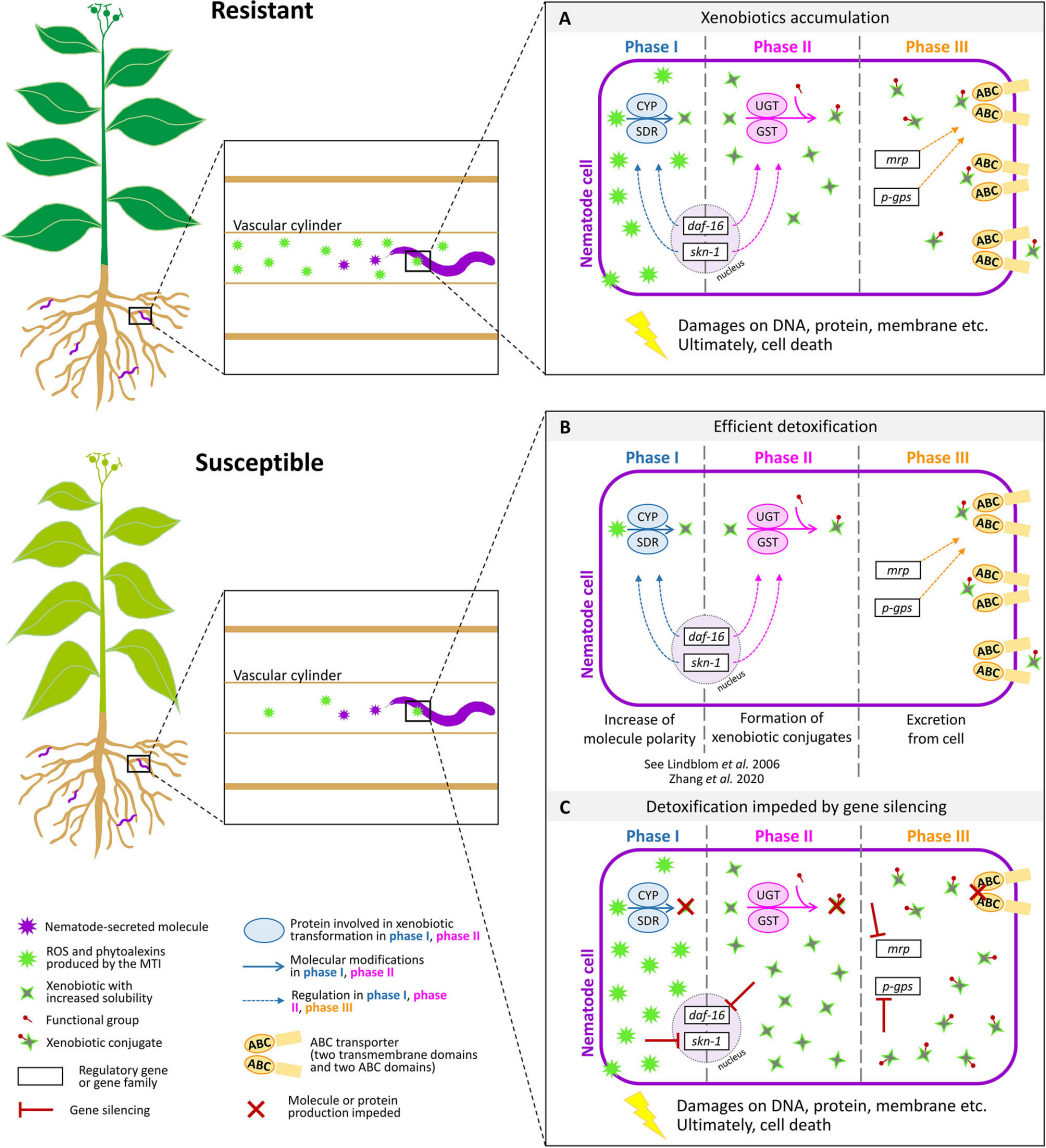
Response of plant-parasitic nematodes in resistant and susceptible plants. Resistant plants produce reactive oxygen species (ROS) and phytoalexins through the microbe- or pathogen-associated molecular patterns (MAMPs or PAMPs), in response to the detection of nematode-secreted molecules. Susceptible plants produce less compounds to defend the nematode presence. Plant-parasitic nematodes have evolved xenobiotic metabolic pathways to counteract the cytotoxic defense response of plants **(A)**. During phase I of xenobiotic metabolism, the polarity and solubility of xenobiotics is increased, often by oxidation, reduction, or hydrolysis reactions. In phase II, a functional group is added to form xenobiotic conjugates. The production of proteins involved in phase I and II is orchestrated by two transcription factors, *daf-16* and *skn-1*. In phase III, ABC transporters excrete xenobiotic conjugates from the cell. The expression of genes coding for ABC transporters is regulated by gene families such as *MRP* and *P-GPS* **(B)**. Silencing genes involved in xenobiotic detoxification compromise nematode cell survival. The model proposes that silencing genes involved in phase III would lead to the impediment of the production of ABC transporters and thus to a lethal accumulation of xenobiotic conjugates that could not be excreted from the cell **(C)**. ABC, ATP-binding cassette; CYP, cytochrome P450; GST, glutathione S-transferase; SDR, short-chain dehydrogenase; UGT, UDP-glucuronosyl or UDP-glycosyltransferase; *daf-16*, dauer formation 16; s*kn-1*, skinhead transcription factor-1; *mrp*, multi-drug resistance protein; MTI, MAMP-triggered immunity; *p-gps*, P-glycoproteins. Credits: root shape was derived from Figure 1 in Schouteden et al. (2015).

A strongly conserved ATP-binding motif and highly conserved functional arrangement in membranes are the signature keys of the ABC transporters present in different organisms, from bacteria to humans (Higgins, 1992; Childs and Ling, 1994; Linton and Higgins, 1998). With 60 genes, ABC transporters constitute the largest family of transporters in the genome of *Caenorhabditis elegans*, where they have been shown to be associated with drug resistance (Sheps et al., 2004; Pohl et al., 2011; Ardelli, 2013). Furthermore, elevated expression of ABC transporter genes has been reported in animal-parasitic nematodes (APNs) in association with resistance to drugs such as ivermectin and macrocyclic lactones (Xu et al., 1998; Prichard and Roulet, 2007; Stitt et al., 2011). However, very few reports on the role of ABC transporters in PPNs exist. The genome of the PPN *Bursaphelenchus xylophilus* has 106 ABC transporters, which is almost double the number found in the genome of *C. elegans* and about three times more than what is found in the genome of *M. incognita* (Kikuchi et al., 2011). Upregulation of *Bursaphelenchus* ABC transporter genes in response to a α-pinene, a monoterpene produced by plants in response to pathogen attack, has been found recently (Li et al., 2019). Diao et al., 2020 investigated the multi-drug-resistant protein coding (MDR) genes in *B. xylophilus* with a focus on screening nematicides (emamectin benzoate, avermectin, and matrine) for the control of these devastating nematodes and found that members of the MDR gene family encode the ABC transporter and the ABC transporter transmembrane region. Fu et al. (2020) recently compared gene expression patterns between hydrated and 24-h desiccated nematodes of the foliar nematode *Aphelenchoides fragariae*. This study shows differential expression of detoxification genes, including the *pgp-14*-like multi-drug resistance protein (MRP/PGP), which is part of the ABC transporter system (Figure 1).

Interactions of *G. pallida* with its natural host *Solanum tuberosum* and a resistant plant *Solanum sisymbriifolium* was investigated previously (Kooliyottil et al., 2016, 2019). A hypersensitive response (HR) was evident as early as 24 h post infestation in the root cells of *S. sisymbriifolium* (Kooliyottil et al., 2016). Transcriptome analysis of *G. pallida* juveniles isolated from resistant *S. sisymbriifolium* or the susceptible host, potato, 24 h post infestation showed expression of several genes related to the xenobiotic metabolism (Kooliyottil et al., 2019). *G. pallida* isolated from *S. tuberosum* and *S. sisymbriifolium* showed that there were 18 *G. pallida* ABC transporters (GPLIN_000375400, GPLIN_001624000, GPLIN_000079800, GPLIN_000593000, GPLIN_000607700, GPLIN_000662100, GPLIN_000762600, GPLIN_000764700, GPLIN_000165600, GPLIN_001513300, GPLIN_000934100, GPLIN_001038000, GPLIN_001558100, GPLIN_001072100, GPLIN_000043700, GPLIN_001213800, GPLIN_001600700, and GPLIN_000055000) expressed during plant infection (Kooliyottil et al., 2019). Although not significantly different, six ABC transporter genes were over-expressed when *G. pallida* infected resistant plant *S. sisymbriifolium*, and four were over-expressed when infecting susceptible *S. tuberosum*, and the rest were expressed with similar values when infecting both plant species (Kooliyottil et al., 2019).

## Prospects: Nematode ABC Transporters as Potential Target to Control Plant-Parasitic Nematodes

Transcriptome information of PPNs isolated from resistant plant species is scanty. Most of the available PPN transcriptome information comes from studies on susceptible plant species. Data obtained from nematode-infected resistant plants is scanty and does not provide evidence on expression of ABC transporters (Shukla et al., 2018; Cotton et al., 2014). Although our results (Kooliyottil et al., 2019) are not showing statistically significant difference in *G. pallida* ABC transporter expression when infecting a resistant or susceptible plant species, the expression of genes coding for ABC transporter proteins suggests that they play a role in plant infection. Genes associated with xenobiotic biodegradation pathways were upregulated when *G. pallida* infected resistant *S. sisymbriifolium*. This attribute toward the nematode's defense response to secondary metabolites is induced by *S. sisymbriifolium* upon invasion (Kooliyottil et al., 2019). This is evident from our previous study that the juveniles were unable to detoxify the secondary metabolites, leading to death within the root during the early stages of parasitism (Kooliyottil et al., 2016). The hostile environment in a resistant plant cell could be compared to the chemicals/drugs used for treatment against APNs. As a response to drugs, over-expression of ABC transporters was reported in several nematodes including *C. elegans* (Ardelli, 2013). Furthermore, when ABC transporter genes *mrp-1* and *pgp-1* were silenced in *C. elegans*, the sensitivity toward the drugs or heavy metal ions was increased as compared to the wild type (Broeks et al., 1996). Increased expression of ABC transporter system genes *MRP* and *PGP* in *C. elegans* resistant to ivermectin was reported by James and Davey (2009).

Several reports are available on the xenobiotic metabolism in PPNs, and these genes have been successfully used for gene silencing research with a focus on developing PPN-resistant crop varieties. An investigation about ABC transporters in PPNs, especially when interacting with resistant plant species, may provide useful information about how nematodes are able to overcome plant defenses (Figure 1). Considering the existing knowledge on the importance of ABC transporters in APNs, characterization of ABC transporter genes may contribute to the identification of gene targets for silencing and may provide novel strategies for PPN control. Silencing ABC transporters in APNs has proven effective and is considered as a great tool to control these parasites. We propose further research to determine the role of ABC transporters and other genes involved in xenobiotic response of PPNs to stress conditions as may be encountered in resistant plants. Understanding the role and mechanisms of ABC transporters in PPNs will be helpful to identify the strategies for achieving sustainable pest control and may even facilitate development of PPN-resistant plants.

## Author Contributions

RK developed the concept. RK and KR analyzed the data. RK, KR, NS, and L-MD contributed to discussions and writing the manuscript. All authors contributed to the article and approved the submitted version.

## Conflict of Interest

The authors declare that the research was conducted in the absence of any commercial or financial relationships that could be construed as a potential conflict of interest.
